# Supplementary material for: Genetic variants determine intrafamilial variability of SARS-CoV-2 clinical outcomes in 19 Italian families
Source: PLoS One. 2022 Oct 13;17(10):e0275988. doi: 10.1371/journal.pone.0275988 (PMC9560599; doi:10.1371/journal.pone.0275988)
Supplement: S1 Table — (DOCX) [file pone.0275988.s001.docx]

**S1 Table. List of genes in the customized panel.**

| **Gene** | **RefSeq** |
| --- | --- |
| *ACE2* | NM_021804 |
| *ADAR* | NM_001111.4 |
| *BIRC2* | NM_001166.4 |
| *BIRC3* | NM_001165.4 |
| *CCR9* | NM_031200 |
| *CD209* | NM_021155.3 |
| *CD40LG* | NM_000074 |
| *CFL1* | NM_005507.2 |
| *CFL2* | NM_138638.4 |
| *CLEC4M* | NM_014257.4 |
| *CXCR3* | NM_001142797 |
| *CXCR6* | NM_006564.1 |
| *DDX58* | NM_014314.3 |
| *FOXP3* | NM_014009 |
| *FYCO1* | NM_024513.3 |
| *IFIH1* | NM_022168.3 |
| *IFITM3* | NM_021034.2 |
| *IFNAR1* | NM_000629.2 |
| *IFNAR2* | NM_207585.2 |
| *IL1A* | NM_000575.4 |
| *IL1B* | NM_000576 |
| *IL6* | NM_000576 |
| *IRF7* | NM_004031.2 |
| *LZTFL1* | NM_020347 |
| *MAVS* | NM_020746.4 |
| *NEU1* | NM_000434 |
| *NFKB1* | NM_003998.3 |
| *NLRP1* | NM_033004.3 |
| *NLRP3* | NM_004895.4 |
| *SLC6A20* | NM_020208.3 |
| *STAT1* | NM_007315.3 |
| *STAT2* | NM_005419 |
| *TLR2* | NM_003264.4 |
| *TLR3* | NM_003265.2 |
| *TLR4* | NM_138554.4 |
| *TLR7* | NM_016562.3 |
| *TLR8* | NM_016610.3 |
| *TMPRSS2* | NM_001135099.1 |
| *TNF* | NM_000594 |
| *TRAF3* | NM_003300 |
| *TRAF6* | NM_004620.3 |
| *XCR1* | NM_001024644 |
