# Supplementary material for: Genetic variants determine intrafamilial variability of SARS-CoV-2 clinical outcomes in 19 Italian families
Source: PLoS One. 2022 Oct 13;17(10):e0275988. doi: 10.1371/journal.pone.0275988 (PMC9560599; doi:10.1371/journal.pone.0275988)
Supplement: S2 Table — a Human GRCh37/hg19; b Minor Allele Frequency. Red: variants already reported as of risk by the COVID-19 HGI in the UCSC Genome Browser. (DOCX) [file pone.0275988.s002.docx]

**S2 Table: Familial segregation of all risk variants in families with positive SARS-CoV-2 test.**

| **Family N°** | **Position ^a^** | **Ref** | **Alt** | **Gene** | **AA change** | **gnomAD MAF % ^b^** | **dbSNP** | **COVID-19 Host Genetics Initiative** |
| --- | --- | --- | --- | --- | --- | --- | --- | --- |
| 4 | chr20:3838441 | C | G | MAVS | p.Gln93Glu | 28 | rs17857295 |  |
| 5 | chr21:34614255 | T | G | IFNAR2 | p.Phe10Val | 37,5 | rs1051393 | A2, B2, C2 |
| 5 | chr21:34634878 | G | A | IFNAR2 | p.Ala285Thr | 38 | rs1131668 | A2, B2 |
| 5 | chr21:42879909 | C | A | TMPRSS2 | p.Gly8Val | 35,1 | rs75603675 |  |
| 5 | chr4:187004074 | C | T | TLR3 | p.Leu412Phe | 27,5 | rs3775291 |  |
| 6 | chr1:247587343 | G | A | NLRP3 | p.Val200Met | 0,83 | rs121908147 |  |
| 8 | ch1:154574820 | T | C | ADAR | p.Lys384Arg | 99,9 | rs1466731 |  |
| 8 | chr17:5425077 | T | C | NLRP1 | p.Met1184Val | 45,1 | rs11651270 |  |
| 8 | chr2:163128824 | T | C | IFIH1 | p.His843Arg | 67,6 | rs3747517 |  |
| 8 | chr20:3843027 | C | A | MAVS | p.Gln198Lys | 14,5 | rs7262903 |  |
| 8 | chr3:46009864 | C | G | FYCO1 | p.Gly321Ala | 56,5 | rs3733100 |  |
| 8 | chr3:46010077 | C | T | FYCO1 | p.Arg250Gln | 84,6 | rs4683158 |  |
| 9 | chr1:154574820 | T | C | ADAR | p.Lys384Arg | 99,9 | rs1466731 |  |
| 9 | chr2:163124051 | C | T | IFIH1 | p.Ala946Thr | 49,7 | rs1990760 |  |
| 9 | chr2:163128824 | T | C | IFIH1 | p.His843Arg | 67,6 | rs3747517 |  |
| 9 | chr20:3844929 | C | T | MAVS | p.Arg218Cys | 10,9 | rs45437096 |  |
| 9 | chr3:46010077 | C | T | FYCO1 | p.Arg250Gln | 84,6 | rs4683158 |  |
| 10 | chr21:34614255 | T | G | IFNAR2 | p.Phe10Val | 37,5 | rs1051393 | A2, B2, C2 |
| 10 | chr21:34634878 | G | A | IFNAR2 | p.Ala285Thr | 38 | rs1131668 | A2, B2 |
| 10 | chr3:46007823 | GTT | TTC | FYCO1 | p.Asn1001Glu | 12 | rs13079478, rs13059238 | A2,B2,C2 |
| 10 | chr3:46008820 | C | T | FYCO1 | p.Ser669Asn | 0,15 | rs141155944 |  |
| 10 | chr3:46008841 | G | A | FYCO1 | p.Ser662Phe | 0,15 | rs150785981 |  |
| 10 | chr3:46009864 | C | G | FYCO1 | p.Gly321Ala | 56,5 | rs3733100 |  |
| 10 | chr3:46010077 | C | T | FYCO1 | p.Arg250Gln | 84,6 | rs4683158 |  |
| 12 | chr2:113537223 | C | A | IL1A | p.Ala114Ser | 26,8 | rs17561 |  |
| 12 | chr3:45837886 | G | C | SLC6A20 | p.Ala9Gly | 11,7 | rs2271615 | C1, C2 |
| 12 | chrX:12903659 | A | T | TLR7 | p.Gln11Leu | 17,9 | rs179008 |  |
| 14 | chr3:46007823 | GTT | TTC | FYCO1 | p.Asn1001Glu | 12 | rs13079478 rs13059238 | A2,B2,C2 |
| 14 | chr3:46009487 | G | A | FYCO1 | p.Arg447Cys | 12,1 | rs33910087 | A2,B2,C2 |
| 14 | chr3:46009864 | C | G | FYCO1 | p.Gly321Ala | 56,5 | rs3733100 |  |
| 16 | chrX:12924826 | A | G | TLR8 | p.Met1? | 30,5 | rs3764880 |  |
| 18 | chr14:103342049 | T | C | TRAF3 | p.Met129Thr | 32,8 | rs1131877 |  |
| 19 | chr11:614318 | T | C | IRF7 | p.Lys192Glu | 26,1 | rs1061502 |  |
