# Supplementary material for: Genetic variants determine intrafamilial variability of SARS-CoV-2 clinical outcomes in 19 Italian families
Source: PLoS One. 2022 Oct 13;17(10):e0275988. doi: 10.1371/journal.pone.0275988 (PMC9560599; doi:10.1371/journal.pone.0275988)
Supplement: S3 Table — a Human GRCh37/hg19; b Minor Allele Frequency. Red: variants already reported as of risk by the COVID-19 HGI in the UCSC Genome Browser. Blue: variants already reported as of protection by the COVID-19 HGI in the UCSC Genome Browser. (DOCX) [file pone.0275988.s003.docx]

**S3 Table: Familial segregation of all protective variants in families with negative SARS-CoV-2 test.**

| **Family N°** | **Position ^a^** | **Ref** | **Alt** | **Gene** | **AA change** | **GnomAD MAF % ^b^** | **dbSNP** | **COVID-19 Host Genetics Initiative** |
| --- | --- | --- | --- | --- | --- | --- | --- | --- |
| 5 | chr21:42852497 | C | T | TMPRSS2 | p.Val197Met | 24,9 | rs12329760 | B2 |
| 6 | chr17:5442790 | C | T | NLRP1 | p.Val939Met | 1,4 | rs61754791 |  |
| 6 | chr17:5485367 | A | T | NLRP1 | p.Leu155His | 36,2 | rs12150220 |  |
| 10 | chr11:320805 | G | T | IFITM3 | p.His3Gln | 4,1 | rs1136853 |  |
| 10 | chr11:613208 | T | C | IRF7 | p.Gln425Arg | 25,8 | rs1131665 |  |
| 10 | chr11:614318 | T | C | IRF7 | p.Lys192Glu | 26,1 | rs1061502 |  |
| 10 | chr21:34715699 | G | C | IFNAR1 | p.Val168Leu | 18 | rs2257167 |  |
| 10 | chr21:42852497 | C | T | TMPRSS2 | p.Val197Met | 24,9 | rs12329760 | B2 |
| 13 | chr17:5425077 | T | C | NLRP1 | p.Met1184Val | 45,1 | rs11651270 |  |
| 18 | chr11:613208 | T | C | IRF7 | p.Gln425Arg | 25,8 | rs1131665 |  |
| 18 | chr11:614318 | T | C | IRF7 | p.Lys192Glu | 26,1 | rs1061502 |  |
| 18 | chr20:3844929 | C | T | MAVS | p.Arg218Cys | 10,9 | rs45437096 |  |
| 18 | chr21:34614255 | T | G | IFNAR2 | p.Phe10Val | 37,5 | rs1051393 | A2, B2, C2 |
| 18 | chr21:34634878 | G | A | IFNAR2 | p.Ala285Thr | 38 | rs1131668 | A2, B2 |
| 18 | chr21:42866296 | T | C | TMPRSS2 | p.Thr112Thr | 10 | rs3787950 |  |
| 18 | chr21:42866297 | G | A | TMPRSS2 | p.Thr112Ile | 0,7 | rs61735793 |  |
| 18 | chr21:42879909 | C | A | TMPRSS2 | p.Gly8Val | 35,1 | rs75603675 |  |
