# Supplementary material for: Genetic variants determine intrafamilial variability of SARS-CoV-2 clinical outcomes in 19 Italian families
Source: PLoS One. 2022 Oct 13;17(10):e0275988. doi: 10.1371/journal.pone.0275988 (PMC9560599; doi:10.1371/journal.pone.0275988)
Supplement: S4 Table — a Human GRCh37/hg19; b Minor Allele Frequency. Red: variants already reported as of risk by the COVID-19 HGI in the UCSC Genome Browser. (DOCX) [file pone.0275988.s004.docx]

**S4 Table: Familial segregation of all variants in families with severe COVID-19.**

| **Family N°** | **Position ^a^** | **Ref** | **Alt** | **Gene** | **AA change** | **GnomAD MAF % ^b^** | **dbSNP** | **COVID-19 Host Genetics Initiative** |
| --- | --- | --- | --- | --- | --- | --- | --- | --- |
| 1 | chr11:613208 | T | C | IRF7 | p.Gln425Arg | 25,8 | rs1131665 |  |
| 1 | chr11:614318 | T | C | IRF7 | p.Lys192Glu | 26,1 | rs1061502 |  |
| 2 | chr4:187004074 | C | T | TLR3 | p.Leu412Phe | 27,5 | rs3775291 |  |
| 3 | chr17:5442790 | C | T | NLRP1 | p.Val939Met | 1,4 | rs61754791 |  |
| 4 | chr20:3838441 | C | G | MAVS | p.Gln93Glu | 28 | rs17857295 |  |
| 6 | chr11:613978 | C | T | IRF7 | p.Gly260Arg | 0,069 | rs201379782 |  |
| 6 | chr11:614799 | C | T | IRF7 | p.Arg144Gln | 0,06 | rs201036875 |  |
| 6 | chr11:102248377 | C | T | BIRC2 | p.Ala506Val | 4,8 | rs34510872 |  |
| 6 | chr19:7831628 | G | A | CLEC4M | p.Asp291Asn | 26,5 | rs2277998 |  |
| 6 | chr21:42866296 | T | C | TMPRSS2 | p.Thr112Thr | 10 | rs3787950 |  |
| 6 | chr21:42879909 | C | A | TMPRSS2 | p.Gly8Val | 35,1 | rs75603675 |  |
| 6 | chr3:45801393 | T | C | SLC6A20 | p.Ile529Val | 0,72 | rs61731475 |  |
| 6 | chr3:45869972 | C | T | LZTFL1 | p.Asp246Asn | 7,4 | rs1129183 |  |
| 6 | chr9:32500832 | C | T | DDX58 | p.Arg71His | 0,9 | rs72710678 |  |
| 6 | chr9:120475302 | A | G | TLR4 | p.Asp299Gly | 6,1 | rs4986790 |  |
| 6 | chr9:120475602 | C | T | TLR4 | p.Thr399Ile | 5,6 | rs4986791 |  |
| 7 | chr19:7831628 | G | A | CLEC4M | p.Asp291Asn | 26,5 | rs2277998 |  |
| 8 | chrX:12903659 | A | T | TLR7 | p.Gln11Leu | 17,9 | rs179008 |  |
| 8 | chrX:12924826 | A | G | TLR8 | p.Met1? | 30,5 | rs3764880 |  |
| 13 | chr3:45837886 | G | C | SLC6A20 | p.Ala9Gly | 11,7 | rs2271615 | C1, C2 |
| 13 | chr3:45987980 | G | A | CXCR6 | p.Glu3Lys | 4,4 | rs2234355 |  |
| 13 | chr3:46007846 | C | T | FYCO1 | p.Glu994Lys | 2,6 | rs34801630 |  |
| 16 | chr9:32480251 | A | T | DDX58 | p.Asp580Glu | 10 | rs17217280 |  |
| 19 | chr4:103534740 | - | A | NFKB1 | c.2749+11dupA | 6,5 | rs148268461 |  |
