# Supplementary material for: Genetic variants determine intrafamilial variability of SARS-CoV-2 clinical outcomes in 19 Italian families
Source: PLoS One. 2022 Oct 13;17(10):e0275988. doi: 10.1371/journal.pone.0275988 (PMC9560599; doi:10.1371/journal.pone.0275988)
Supplement: S5 Table — R: Risk variant; S: Severity variant; U: Unclassified; /: Absent. a Human GRCh37/hg19; b Minor Allele Frequency. (DOCX) [file pone.0275988.s005.docx]

**S5 Table: All rare variants (MAF<1%) identified in subjects of our families.**

| **Position ^a^** | **Ref** | **Alt** | **Gene** | **AA change** | **gnomAD MAF % ^b^** | **dbSNP** | **CADD score** | **VarSome tools in silico prediction** | **N° Positive** | | | **N° Negative** | **Patient ID** |
| --- | --- | --- | --- | --- | --- | --- | --- | --- | --- | --- | --- | --- | --- |
|  |  |  |  |  |  |  |  |  | **Severe** | **Mild** | **Asintomatic** |  |  |
| chr11:102248406 | A | G | **BIRC2** | p.Lys516Glu | 0,15 | rs61754131 | 27 | 8/12 damaging |  | 1 | 2 |  | 14M; 14S1; 14GF |
| chr11:102239127 | A | G | **BIRC2** | p.Asn405Ser | 0,002 | rs187423268 | 23 | 11/12 tolerated |  | 3 | 1 |  | 15M; 15S1;15S2;15S3 |
| chr11:102201850 | G | A | **BIRC3** | p.Arg401Lys | 0,5 | rs17881197 | 23 | 11/11 tolerated |  |  |  | 2 | 1G; 14F |
| chr3:45942554 | A | G | **CCR9** | p.Ile92Val | 0,56 | rs45530037 | 0 | 11/12 tolerated |  |  | 1 | 2 | 6S1; 6M; 6S2 |
| chr19:7812230 | A | G | **CD209** | p.Leu23Pro | 0,002 | rs765954889 | 3 | 11/12 tolerated | 1 |  |  | 1 | 10F; 10S2 |
| chr9:32489407 | T | C | **DDX58** | p.Asn245Ser | 0,22 | rs141808660 | 11 | 10/11 tolerated |  |  |  | 1 | 1G |
| chr9:32500832 | C | T | **DDX58** | p.Arg71His | 0,9 | rs72710678 | 23 | 11/17 tolerated | 1 | 1 | 1 | 1 | 6F;6S3; 14M; 14GM |
| chr9:32467918 | G | A | **DDX58** | p.Pro676Leu | 0,004 | rs374942642 | 23 | 12/17 tolerated |  | 1 | 1 |  | 12A; 12GM |
| chr3:46008820 | C | T | **FYCO1** | p.Ser669Asn | 0,15 | rs141155944 | 14 | 11/12 tolerated | 1 |  |  |  | 10F |
| chr3:46008841 | G | A | **FYCO1** | p.Ser662Phe | 0,15 | rs150785981 | 24 | 7/12 damaging | 1 |  |  |  | 10F |
| chr3:45965190 | G | A | **FYCO1** | p.Thr1440Ile | 0,3 | rs41289612 | 22 | 10/12 tolerated |  |  | 1 |  | 15M |
| chr2:163124596 | C | T | **IFIH1** | c.2807+1G>A | 0,6 | rs35732034 | 33 | 6/6 damaging | 2 |  |  | 1 | 1F; 1U; 3F |
| chr2:163133396 | G | A | **IFIH1** | p.Thr702Ile | 0,2 | rs72650663 | 3 | 12/12 tolerated | 1 |  |  | 1 | 1F; 1U |
| chr2:163134090 | C | A | **IFIH1** | p.Glu627Ter | 0,31 | rs35744605 | 38 | 5/5 damaging |  | 1 |  | 1 | 2M; 2S2 |
| chr2:163128755 | G | A | **IFIH1** | p.Pro866Leu | 0,04 | rs200833729 | 24 | 14/19 tolerated |  | 1 | 1 | 1 | 5M; 5GM; 5S1; |
| chr2:163124024 | G | C | **IFIH1** | p.Gln955Glu | 0,096 | rs144455277 | 12 | 11/12 tolerated |  |  |  | 1 | 6S2 |
| chr2:163136505 | C | G | **IFIH1** | c.1641+1G>C | 0,67 | rs35337543 | 32 | 6/6 damaging |  | 1 |  |  | 9F |
| chr2:163133202 | T | - | **IFIH1** | p.Thr767HisfsTer17 | 0,0057 | rs759430873 | 34 | 1/1 damaging |  |  | 1 |  | 14GF |
| chr21:34635293 | C | T | **IFNAR2** | p.Pro346Ser | 0,0024 | rs148519830 | 1 | 11/11 tolerated |  | 2 |  |  | 4M;4S2 |
| chr21:34625037 | C | G | **IFNAR2** | p.Thr204Arg | 0,5 | rs147496374 | 23 | 9/18 tolerated |  | 1 |  | 3 | 11GM; 11U; 15MM; 15SS |
| chr21:34625019 | A | G | **IFNAR2** | p.Asp198Gly | 0,3 | rs200536427 | 24 | 9/12 tolerated |  | 1 | 1 |  | 12M; 12GM |
| chr2:113590374 | C | T | **IL1B** | p.Glu111Lys | 0,01 | rs201772036 | 4 | 12/12 tolerated |  | 1 |  |  | 8GM |
| chr7:22767134 | C | A | **IL6** | p.Pro31Thr | 0,2 | rs142759801 | 3 | 10/11 tolerated |  |  |  | 1 | 14F |
| chr11:613978 | C | T | **IRF7** | p.Gly260Arg | 0,069 | rs201379782 | 0 | 11/12 tolerated | 1 |  |  | 1 | 6F;6M |
| chr11:614799 | C | T | **IRF7** | p.Arg144Gln | 0,06 | rs201036875 | 12 | 11/12 tolerated | 1 |  |  | 1 | 6F;6M |
| chr11:615129 | C | T | **IRF7** | p.Asp64Asn | - | - | 22 | 10/12 damaging | 1 |  |  | 1 | 7F; 7S2 |
| chr6:31829865 | C | G | **NEU1** | p.Gly88Ala | 0,75 | rs34712643 | 27 | 8/10 damaging |  |  | 1 |  | 14GF |
| chr4:103518700 | A | G | **NFKB1** | p.Met507Val | 0,97 | rs4648072 | 8 | 11/11 tolerated | 1 | 1 |  | 2 | 10F; 10S1;12F;12S1 |
| chr4:103518782 | G | A | **NFKB1** | p.Arg534His | 0,053 | rs150281816 | 26 | 8/12 damaging |  |  |  | 1 | 10M |
| chr17:5462129 | G | T | **NLRP1** | p.Phe629Leu | 0,15 | rs149035689 | 26 | 10/17 damaging |  | 3 | 1 | 2 | 4M; 4S1;4S2; 5M; 5GM; 5S1; |
| chr1:247587343 | G | A | **NLRP3** | p.Val200Met | 0,83 | rs121908147 | 0 | 9/10 tolerated | 1 |  | 1 |  | 6F; 6S1 |
| chr1:247582310 | G | A | **NLRP3** | p.Val72Met | 0,072 | rs117287351 | 17 | 8/10 tolerated |  |  |  | 1 | 18M |
| chr3:45812898 | A | G | **SLC6A20** | p.Phe249Ser | 0,1 | rs147760034 | 28 | 11/12 damaging | 1 | 1 | 1 | 1 | 1M; 1S1;1S2;1S3 |
| chr3:45801393 | T | C | **SLC6A20** | p.Ile529Val | 0,72 | rs61731475 | 18 | 9/11 tolerated | 1 | 1 | 2 |  | 6F; 12A; 12M; 12GM |
| chr12:56737251 | C | A | **STAT2** | p.Gln826His | 0,8 | rs2229363 | 16 | 9/11 tolerated |  | 3 | 1 |  | 15M; 15S1;15S2;15S3 |
| chr4:187004500 | C | T | **TLR3** | p.Pro554Ser | 0,04 | rs121434431 | 23 | 9/12 damaging |  | 1 | 1 |  | 5M; 5GF |
| chr4:187003729 | C | G | **TLR3** | p.Leu297Val | 0,15 | rs35311343 | 23 | 7/11 damaging |  | 1 | 2 |  | 12A; 12M; 12S1 |
| chr4:187003759 | T | G | **TLR3** | p.Tyr307Asp | 0,3 | rs5743317 | 15 | 10/11 tolerated |  |  |  | 1 | 15MM |
| chrX:12904292 | T | A | **TLR7** | p.Val222Asp | 0,2 | rs55907843 | 5 | 10/11 tolerated |  | 2 | 1 |  | 15M; 15S2;15S3 |
| chr21:42879910 | C | G | **TMPRSS2** | p.Gly8Arg | 0,59 | rs200291871 | 10 | 9/9 tolerated | 1 |  |  | 1 | 10F; 10S2 |
| chr21:42866297 | G | A | **TMPRSS2** | p.Thr112Ile | 0,7 | rs61735793 | 9 | 11/11 tolerated |  | 3 | 1 | 3 | 15M;15S1;15S2;15S4;18M;18S1;18S2 |
| chr14:103336572 | G | A | **TRAF3** | p.Ala12Thr | 0,03 | rs139127242 | 0 | 12/12 tolerated |  | 1 | 1 |  | 14M;14GM |
| chr11:36512113 | C | A | **TRAF6** | p.Val282Phe | 0,002 | rs376933557 | 17 | 11/12 tolerated |  |  |  | 2 | 13F;13S2 |
