# Supplementary material for: Genetic variants determine intrafamilial variability of SARS-CoV-2 clinical outcomes in 19 Italian families
Source: PLoS One. 2022 Oct 13;17(10):e0275988. doi: 10.1371/journal.pone.0275988 (PMC9560599; doi:10.1371/journal.pone.0275988)
Supplement: Attachment — Submitted filename: Response to Reviewers.docx [file pone.0275988.s006.docx]

*Dear Editor*,

We are grateful to the reviewers for the comments and criticisms and we have addressed them specifically.

Response to reviewer


- Regarding the global statistical analysis: as the authors acknowledged, the sample size was small for this analysis and some associations could be lost due to lack of statistical power. This needs to be better discussed in the Discussion section as a limitation of the study.

**A: We included in the text this aspect in the Discussion section (page 27 lines 463-465).**

- Page 14, lines 187-188: What did the authors mean by gene mutation rate? Frequency of each variant? If yes, please correct the information in the text since mutation rate is not the same as the frequency of individual variants.

**A: We don’t refer to allele frequency, but the "mutation rate" means the number of variants identified in that gene in our population, in other words how much that gene was mutated in different groups of individuals (*i.e*. gene specific mutation burden). We specified in all Tables the “gnomAD %” as the “gnomAD MAF %”. We have specified this information in the text (page 12 line 192).**

- There are 8 tables (plus 2 figures). Some tables are unnecessary. Table 2 could be added in the Supplementary material and table 7 could be excluded and the information only written in the main text.

**A: We agree with the reviewer. We moved Table 2 in the Supplementary Materials (new Suppl. Table 1) and we excluded Table 7. All numbers of the tables were modified: 6 tables (plus 2 figures) in the manuscript and 5 tables in Supplementary tables.**

- Table 8 (Statistical global analysis of gene variants): This table could be better described and formatted. Are the frequencies shown for the minor alleles? The authors could add OR for each variant. Please check the frequency of the most mutated gene in positive individuals (STAT2). Positive is 0.62% only? And for what variants these frequencies of the most mutated genes in positive and negative individuals are shown?

**A: We agree with the reviewer. We added in the Table 8 (now table 6 in the revised version) the odds ratios with the confidence intervals for each results.**

**We better formatted and described the table in Results of Global analysis section (page 21 lines 312-338).**

**Moreover, the percentage refers to individuals carrying that specific variant in the study population, not the MAF (minor allele frequency). The MAF of each the variants were reported in the previously tables of the familial segregation (Tables 2-5).**

***STAT2* was the only gene whose variants were detected exclusively in positive individuals (*i.e.* 2 variants in 2 patients out of mutated).**

- Discussion (page 30, line 432): “…whereas it showed a trend for NLRP1 (p=0.093) as a protective gene (data not shown)”. The authors declared that all information was included in the paper; thus, these data not shown must be included in the paper.

**A: “Data not shown” refers to the absence of the gene in the table because we included only the data with a significant p-value (0.093 added). We just wanted to highlight the trend of *NRLP1* gene in text (moved to page 26 line 443).**

- The Discussion section is too long. It could be shortened.
**A: As requested, we shorted the discussion.**

- It would be interesting to cite a recent systematic review and meta-analysis published in Plos One regarding the association of genetic variants and COVID-19:
Genetic polymorphisms associated with susceptibility to COVID-19 disease and severity: A systematic review and meta-analysis. Dieter C et al. PLoS One. 2022 Jul 6;17(7):e0270627. doi: 10.1371/journal.pone.0270627.

**A: We have included reference #3**
